# Structural Elucidation of Rift Valley Fever Virus L Protein towards the Discovery of Its Potential Inhibitors

**DOI:** 10.3390/ph15060659

**Published:** 2022-05-25

**Authors:** Mubarak A. Alamri, Muhammad Usman Mirza, Muhammad Muzammal Adeel, Usman Ali Ashfaq, Muhammad Tahir ul Qamar, Farah Shahid, Sajjad Ahmad, Eid A. Alatawi, Ghadah M. Albalawi, Khaled S. Allemailem, Ahmad Almatroudi

**Affiliations:** 1 Department of Pharmaceutical Chemistry, College of Pharmacy, Prince Sattam Bin Abdulaziz University, Al-Kharj 16273, Saudi Arabia; m.alamri@psau.edu.sa; 2 Department of Chemistry and Biochemistry, University of Windsor, Windsor, ON N9B 3P4, Canada; mumirza@uwindsor.ca; 3 3D Genomics Research Center, College of Informatics, Huazhong Agricultural University, Wuhan 430070, China; m.muzammal.adeel@outlook.com; 4 Department of Bioinformatics and Biotechnology, Government College University Faisalabad, Faisalabad 38000, Pakistan; usmancemb@gmail.com (U.A.A.); farahshahid24@gcuf.edu.pk (F.S.); 5 Department of Health and Biological Sciences, Abasyn University, Peshawar 25000, Pakistan; sajjad.ahmad@abasyn.edu.pk; 6 Department of Medical Laboratory Technology, Faculty of Applied Medical Sciences, University of Tabuk, Tabuk 71491, Saudi Arabia; eid.alatawi@ut.edu.sa; 7 Department of Medical Laboratories, College of Applied Medical Sciences, Qassim University, Buraydah 51452, Saudi Arabia; galbalawi080@gmail.com (G.M.A.); aamtrody@qu.edu.sa (A.A.); 8 Department of Laboratory and Blood Bank, King Fahd Specialist Hospital, Tabuk 47717, Saudi Arabia

**Keywords:** RVFV, RdRp, structural modeling, virtual screening, docking, MD simulation

## Abstract

Rift valley fever virus (RVFV) is the causative agent of a viral zoonosis that causes a significant clinical burden in domestic and wild ruminants. Major outbreaks of the virus occur in livestock, and contaminated animal products or arthropod vectors can transmit the virus to humans. The viral RNA-dependent RNA polymerase (RdRp; L protein) of the RVFV is responsible for viral replication and is thus an appealing drug target because no effective and specific vaccine against this virus is available. The current study reported the structural elucidation of the RVFV-L protein by in-depth homology modeling since no crystal structure is available yet. The inhibitory binding modes of known potent L protein inhibitors were analyzed. Based on the results, further molecular docking-based virtual screening of Selleckchem Nucleoside Analogue Library (156 compounds) was performed to find potential new inhibitors against the RVFV L protein. ADME (Absorption, Distribution, Metabolism, and Excretion) and toxicity analysis of these compounds was also performed. Besides, the binding mechanism and stability of identified compounds were confirmed by a 50 ns molecular dynamic (MD) simulation followed by MM/PBSA binding free energy calculations. Homology modeling determined a stable multi-domain structure of L protein. An analysis of known L protein inhibitors, including Monensin, Mycophenolic acid, and Ribavirin, provide insights into the binding mechanism and reveals key residues of the L protein binding pocket. The screening results revealed that the top three compounds, A-317491, Khasianine, and VER155008, exhibited a high affinity at the L protein binding pocket. ADME analysis revealed good pharmacodynamics and pharmacokinetic profiles of these compounds. Furthermore, MD simulation and binding free energy analysis endorsed the binding stability of potential compounds with L protein. In a nutshell, the present study determined potential compounds that may aid in the rational design of novel inhibitors of the RVFV L protein as anti-RVFV drugs.

## 1. Introduction

Rift Valley fever is a hemorrhagic ailment caused by the Rift Valley Viral Infection (RVVI), affecting humans and livestock. Mosquitos are the vectors for this disease. The first case of RVVI was reported in East Africa’s Rift Valley in Kenya, which made it known as RVF. In its first instance, a significant population of farmed sheep was killed from 1930 to 1931. *Aedex* and *cules* are the two common vectors (mosquitoes) transmitting this virus [1]. Post RVVI, flu-like common symptoms have lasted up to seven days. Although most human infections are mild, approximately four percent of the cases have been severe and show symptoms such as Hemorrhagic fever, meningoencephalitis, and ocular type [2]. 

The virus responsible for this disease is Rift Valley Fever Virus (RVFV), a single-stranded RNA virus belonging to the Phenuviridae Family and the genus Phlebovirus. Rift Valley fever (RVF) is caused by RVFV and has a wide range of clinical symptoms. A mild febrile illness is common in humans and can lead to more severe illnesses such as encephalitis, hemorrhagic fever, and liver disease. Neurological issues including dizziness, paralysis, headaches, hallucinations, vertigo, and delirium are also observed [3]. Up to 10% of RVFV-infected humans experience ophthalmologic complications, including retinal hemorrhaging and photophobia. Sheep, cattle, and goats are more susceptible to RVFV, with a 20–30% mortality rate in adult ruminants and 70% in young animals. Spontaneous abortion in infected pregnant animals is alarmingly high, ranging from 40–100 percent [4]. 

RVF outbreaks are sporadic and linked to meteorological, hydrological, and socioeconomic factors. RVF outbreaks have been reported to produce considerable numbers of infected human cases, which have substantially impacted the healthcare system [5]. According to an analysis by the Center for Disease Control and Prevention (CDC), several RVF outbreaks have occurred in various countries, resulting in hundreds of thousands of human illnesses and human casualties [6]. This disease has spread from the African region to the Arabian to the USA, making it a grave concern to affected regions and the whole world [7]. Epizootics that occur in livestock frequently precede human epidemics. In other words, reducing RVVI in animals is expected to break the transmission cycle and help avoid human sickness [8]. The CDC and the U.S Department of Agriculture (USDA) both classify RVFV as a select agent because of its ability to cause morbidity and mortality and its potential use as a bioterrorism agent [4,9]. Unfortunately, there are no FDA-approved vaccines for humans, but some approved vaccines are available for veterinary use. Similarly, there are no therapeutics available to treat RVFV, requiring additional research in this area.

As per the structure of RVF, it is a high-molecular-weight, single-stranded, linear virion (2–2.5 nm in diameter, 200–300 nm in length, and displaying helical symmetry) enclosed within the ribonucleo-capsid [10]. Three segments of the RVFV genome (S, M, and L-segments) have previously been described as having three separate open reading frames (ORFs) coding for three distinct proteins (viral polymerase, L-protein, and S-protein) [11,12]. The L segment, which encodes only one protein, RNA-dependent RNA polymerase (RdRp), commonly referred to as L protein, is particularly important to our research. The L protein is involved in genomic replication and viral mRNA transcription. The middle region of the L protein contains an RdRp domain, which is found in all RNA viral polymerases and is required for viral RNA production. The RdRp domain retains the six characteristic conserved structural motifs, including PreA/F, A, B, C, D, and E in the central core region [12,13,14,15]. Most of these motifs are located in the palm subdomain and characterize the active site chamber formation [14]. These host mRNAs are employed as primers for viral transcription and suppress viral-RNA-induced immune responses [16]. L protein has an endonuclease domain at its N-terminus that is necessary for cap-snatching, in which it cleaves 50 m7G caps from host mRNAs [17,18]. Viruses translate and reproduce their genome in the cell cytoplasm for survival and growth. During the replication, the virus uses a technique termed cap-snatching, which means taking advantage of two known viral L protein functions: the capability to bind cap structures and cut off the cellular mRNA attached to [19].

The rational drug design process is greatly accelerated by using different computer-aided drug designing applications for in silico drug screening [20,21,22,23,24,25,26,27]. A virtual screening-based drug discovery strategy has been identified as one of the most effective ways to discover and develop new drugs [28]. RVFV L protein was selected for the current study as a potential target for which no drug has been reported so far. Besides evolutionarily conserved motifs in the core region, RdRp has channels/tunnels that link the active site chamber with the exterior and therefore emerge as a potential target for developing anti-viral inhibitors [29,30,31,32], which is evident from the inhibitor design against many deadly viruses such as *Zika virus* (ZIKV) [33,34,35,36], *Japanese encephalitis virus* (JEV) [37], *West Nile virus* (WNV) [38], *Dengue virus* (DENV) [39,40,41], HCV [24,42,43,44], HIV [45], SARS-CoV-2 [25], and most of the drugs have been reported against *Ebola* polymerase L (EBOV) [26]. The current study aimed to use the virtual screening approach to identify potential RVFV L protein inhibitors and inhibitory binding modes of known potent inhibitors, followed by molecular docking analysis to discover novel inhibitors that could be used as potential leads for *RVVI* treatment.

## 2. Results and Discussion

### 2.1. Protein Structural Analysis

Homology modeling is a well-established technique in modern drug discovery, and with more advancement in machine learning approaches, it is now possible to build a homology model with high accuracy even from a template with a low identity. Recent advances in homology modeling have proven their effectiveness as an alternative [46,47] and retrospective analysis, and validate the usefulness of homology modeling in SBVS. [48,49,50,51]. In the current study, the 3D structure of RVFV L protein was predicted by using the homology modeling approach via Modeller.v9.11. It was observed that the crystal structure and cryo-EM structure of severe fever with thrombocytopenia syndrome virus L (SFTSV L) protein (PDB IDs: 6L42 and 6Y6K) were the best hits based on query coverage and percentage identity. 6L42 showed a 34.41% sequence identity and a 91% query coverage during the sequence to structure alignment, while 6Y6K showed a 34.28% sequence identity and a 91% query coverage against the target sequence (Figure S1). Hence, these models were considered the best templates for homology modeling. Chain “A” of both templates were used for downstream structure prediction steps. Studies have suggested that more than 90% confidence indicates that the core model is precise and correct, deviating 2–4 Å in RMSD from the native protein structure [52]. Moreover, a good percentage of identity with maximum coverage between the template and the query sequence indicates a high level of accuracy in the model. The structural superposition/RMSD of the model with the templates is depicted in (Figure S2).

**Figure 1:**
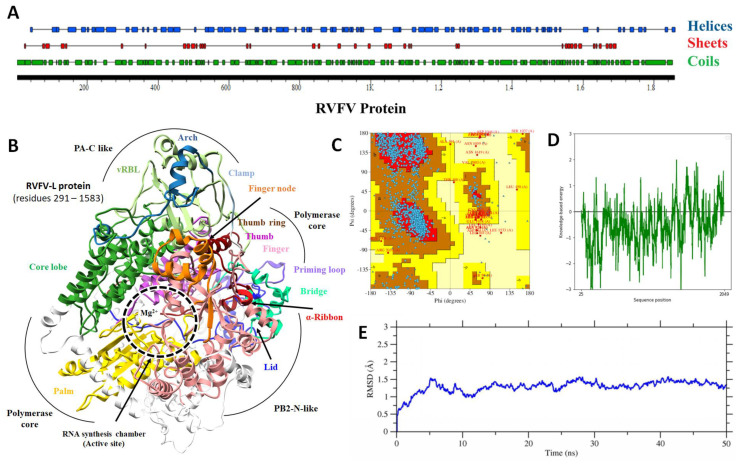
**RVFV L protein structural analysis:** (**A**) Two-dimensional representation of RVFV L protein showing the helices (blue), sheets (red), and coils (green) information (top to bottom). The horizontal black bar is representing the length of a protein. (**B**) Ribbon representation of 3D-model of RVFV L Protein (residues from 291–1583 are highlighted) with divalent cation, presumed to be a magnesium ion (circle Mg^2+^). Each structural component is highlighted with a different color. (**C**) Ramachandran plot contains four quadrants. The 1st and 3rd quadrant indicate the allowed region, while 2nd and 4th show the disallowed region. Blue dots are showing the density of amino acid residues. (**D**) Model energy calculation graph showing the local energy estimation of a model. The *X*-axis indicates the sequence length, while *Y*-axis shows the energy values. (**E**) RMSD of Cα atoms of the RVFV L protein over a period of 50 ns.

Overall, the newly predicted structure showed a pretty stable arrangement of amino-acid residues; apparently, no structural distortion was observed, and a total of 82 helices, 40 sheets, and 118 coils were detected (Figure 1A). Deep structural analysis suggested that the computational model of RVFV protein contains seven domains, i.e., the endoN domain (amino acids 25–205), separated by a linker of span 206–295; the PA-C-like domain from 296 to 762 amino acid; the RdRp core from 763 to 1345 amino acid; the PB2-N-like domain with residues 1346–1571 span; the arm domain contains two spans 1615–1696 and 1811–1932 that are separated by a blocker motif of 1811–1852 residues; the next domain was CBD with the residues 1697–1810, and on the C-terminal, a lariat domain (1933–2049 amino acid) was observed as previously reported [53] (Figure 1B). Some of the RVFV protein model domains were reported as structurally similar to SFTSV-L (severe fever with thrombocytopaenia syndrome virus –L protein) [54], which seems evident because these proteins shared the same values as the *Phenuiviridae* protein family. The orientation of each domain determines the functional specificity of protein and facilitates inter-molecular interactions for domain organization [19,53].

Multiple methods were employed for the validation of the 3D model. The Ramachandran plot indicates that 82.8% of residues were present in the favored region, 15.4% were present in additional allowed regions, and 0.7% were present in disallowed regions (Figure 1C). The presence of 82% of residues in the favored region indicates the high quality of the model. The functional properties of a protein depend mainly on its 3D structure and these properties were analyzed based on secondary structures. It is beneficial to identify secondary structure elements and structural motifs when studying the protein 3D structure [55]. Structural components of L protein were analyzed by PDBsum [56]; results showed the presence of multiple structural components such as 18 Beta-hairpins, 84 helices, 1Psi-loops, 40 strands, 155 Beta-turns, 44 Gamma-turns, 1 Beta-alpha-beta-unit, and 111 helix–helix interactions. Since we detected the 84 helices and 111 helix–helix interactions, such a high number of helices content determines the high chirality of proteins and thus provides structural compactness [57]. The distribution of structural components suggested the potential stability of the newly designed model, which was cross-validated by MD-simulations in subsequent steps. The lowest energy of a model also determines its quality, here we have applied the ProsaWeb server [58] to determine the overall local energy of the RVFV protein model at the amino acid level, results showed that most of the amino acids were showing the lowest energy below the threshold (Figure 1D). A 50 ns MD simulation further evaluated the generated model, and less than a 1.5 Å average RMSD was found that endorsed the reliability of the predicted RVFV-L protein model. An RMSD trajectory plot depicted the overall backbone structure stability of RVFV’s core L protein subdomains (Figure 1E). The backbone Cα-RMSD of RVFV-L fluctuated in the start and converged afterwards. Protein expansion during the start of the simulation probably led to a somewhat larger RMSD of model structure to achieve a more stable conformation. Additionally, the RVFV-L protein homology model was compared with the corresponding template using TM align [59] to identify the likelihood of a similar structural fold (RdRp polymerase chamber) as categorized in SCP/CATH. The TM-algin is the protein structural alignment program for comparing proteins. The TM-align ranked the RVFV-RdRp with up to an 88% similar fold. Reverse template selection by the profunc server [60] (which scans auto-generated templates from the query structure against the most representative structures in PDB using Jess, a fast and accurate 3D-structure search algorithm) also determined SFTSV L (6L42) as the best hits with an E-value of 0.00E+00 and structural similarity of 99.9% (Table S1).

### 2.2. Binding Site Determination 

**Figure 2:**
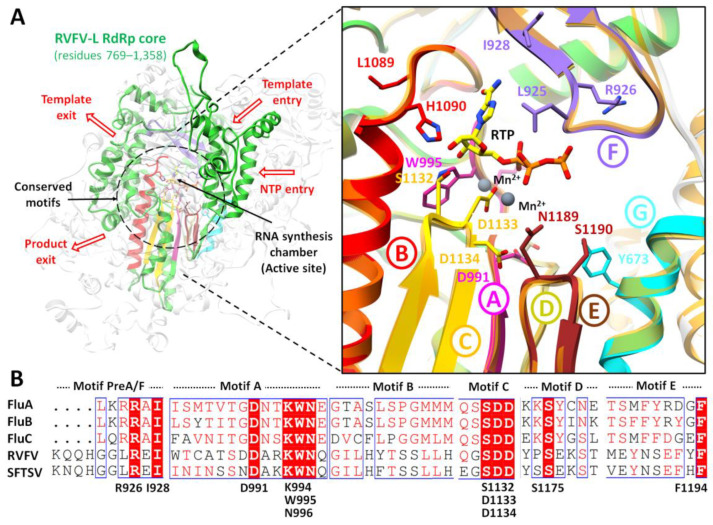
The predicted RVFV-L RdRp active site. (**A**) The RVFV-L RdRp core (residues 769–1358) is highlighted in green with conserved RNA synthesis chamber (active site), organized by conserved structural motifs in distinct colors. The four predicted tunnels are marked with arrows as an entrance (template and NTP entry) into the active site chamber and exit tunnels (template and product exit). The arrangement of structurally conserved RdRp motifs are colored magenta, red, golden, khaki, brown, purple, and cyan for motifs A–F, respectively, and superimposed on SFTSV-L RdRp core (light orange). (**B**) The predicted binding site residues are aligned and highlighted through multiple structure alignment (MSA) in representative motifs. These conserved residues are highlighted in stick representation accordingly. Superposition of the poliovirus elongation complex structure (PDB ID: 3OL8) and foot-and-mouth disease virus in complex with RTP (PDB ID: 2E9R) displays the conformation of RTP (yellow) and positions of the catalytic divalent cations (black spheres). Viral names in MSA are abbreviated as follows: Influenza A (FluA), B (FluB), and C (FluC) virus polymerase, Rift valley fever virus (RVFV), and Severe fever with thrombocytopenia syndrome virus (SFTSV).

The identification and characterization of binding sites of target proteins using various in silico methods are of main consideration for structure-based drug design. For binding site analysis, careful consideration was given to the biological suggestive templates. As RdRps are encoded by a wide range of viruses and play an important role in the replication and transcription of viral RNA [61] and have a conserved RdRp core region including the characteristic conserved finger, palm, and thumb subdomains with conserved 6 structural motifs (motifs A–F), which are essential for polymerase function [31]. Therefore, more in-depth information was extracted from other templates (with low identity) that showed sequence identity in the core region. The overall predicted binding site is diagrammatically illustrated in Figure 2. 

Despite the low sequence identity (<35% identity), the RVFV-L RdRp core (residues 769–1358) displayed characteristic features of the polymerase core domain, which included the RNA synthesis chamber configured by representative RdRp conserved structural motifs connected to the exterior by four channels (for template entry and exit, NTP entry, and product exit) (Figure 2). The active site chamber of the RVFV-L RdRp core is formed by conserved RdRp motifs A-F located in the palm domain as reported in other RNA polymerases, notably in *orthobunyavirus* polymerases [14,62]. For molecular docking, the active site residues were further predicted using COACH and 3DLigandSite, and both servers predicted Arg926, Ile928, Asp991, Lys994, Trp995, Asn996, Gln1084, Ser1132, Asp1133, Asp1134 and Ser1175, and Phe1191 and Phe1194 as common binding site residues. Interestingly, the structural superimposition of RdRp core regions of RVFV-L and SFTSV-L demonstrated these residues to be entirely inside the functionally conserved structural motifs A-F. These include premotif A (motif F) (919-QQHGGL**R**E**I**-928), motif A (991-**D**AR**KWN**-996) with a conserved divalent cation binding residue Asp991, motif B (1084-**Q**GILHYTSSLLH-1095), catalytic signature motif C (1132-**SDD**-1134) located between two β-strands, motif D (1173-YP**S**EKST-1179), and motif E (1186-MEYNS**EF**Y**F**-1194). To further evaluate the binding site for docking simulations, poliovirus elongation complex structure (PDB ID: 3OL8) [63] and foot-and-mouth disease virus in complex with RTP (PDB ID: 2E9R) [64] were superimposed and positions of catalytic divalent cations and conformation of RTP were analyzed. The superimposition revealed that the position of divalent cations was in close connection with functionally conserved aspartates of motif C (Asp1133) and motif A (Asp991) [65,66]. The importance of these aspartates is apparent from a number of mutational studies that uncovered altered polymerase activity in several RdRp viruses [63,67,68,69,70,71,72,73]. The conservation of core residues in these motifs suggests a conserved evolutionary link between RNA polymerase viruses and pinpoints the potential of exploiting the core architecture with other related segmented (−) ssRNA viruses in order to predict their structural/functional features even with a low sequence homology [32,74,75,76]. The docking grid was generated to cover these structural motifs for molecular docking. 

### 2.3. Molecular Docking

**Figure 3:**
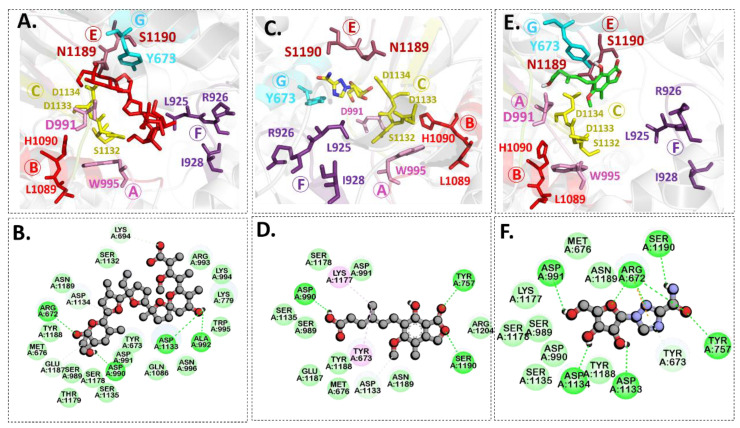
**Binding modes and interaction mechanisms of known L protein inhibitors.** Close-up view into 3D binding mode of (**A**) Monensin, (**C**) Mycophenolic acid, and (**E**) Ribavirin. The main residues that involve in the formation of the active site within the structurally conserved RdRp motifs that are show in in stickes. 2D interaction analysis of (**B**) Monensin, (**D**) Mycophenolic acid, and (**F**) Ribavirin.

Molecular docking is a modeling technique that examines how ligands and receptors fit together and how enzymes interact with ligands [77,78]. The docking computations were done three times, and the compound conformations were sorted by binding energy in kcal/mol. Initially, three known potent inhibitors with distinct chemical structures, namely, Monensin, Mycophenolic acid, and Ribavirin were docked [79]. Monensin inhibits host cell entry by blocking endocytic organelles’ acidification [80]. Mycophenolic acid and Ribavirin act directly by inhibiting the RdRp enzyme or indirectly via the inhibition of cellular enzymes necessary for the biosynthesis of guanine-nucleotide [81,82]. The docking grid-box in Autodock Vina was set up to cover the predicted active site residues within the conserved structural RdRp motifs. As shown in Figure 3, the docked complexes obtained a similar binding orientation within the active chamber RdRp core. For Monensin, it forms hydrogen bonds with the side chains of Arg 672, Asp 990, Asp 1133 (belong to motif C), and Ala 992 (belong to motif A) residues with a binding energy score of −8.5 kcal/mol (Figure 3B). Mycophenolic acid forms hydrogen bonds with residues Arg 1197 and Asn 984 having a binding score of −7.0 kcal/mol (Figure 3D), while Ribavirin forms hydrogen bonds with residues Asp 1134 (belong to motif C), Tyr 757, Ser 1190 (belong to motif E), Arg 672, Asp 991 (a conserved divalent cation binding residue), and Asp 1133 (functionally conserved aspartates of motif C) having a binding score of −6.7 kcal/mol (Figure 3F).

Subsequently, the structure-based virtual screening of the Selleckchem Nucleoside Analogue Library containing 156 diverse compounds against the L protein was performed. Nucleoside or nucleotide analogue is an important class of antiviral therapeutics [83]. It is a powerful tool for dissecting the mechanisms and functions of viral DNA and RNA polymerases [84,85]. This screening selected top-ranking compounds with better binding scores than control compounds for further analysis. The virtual screening results for the top compounds with a binding affinity > −1.5 kcal/mol are shown in Table S2. Table 1 depicts a general overview of the binding energies and residues of control drugs. The docking analysis revealed that A-317491, followed by VER155008, and Khasianine were the best binders among the docked compounds used in this study. The selected compounds exhibited their binding energies in −8.7 kcal/mol to −6.7 kcal/mol. 

**Figure 4:**
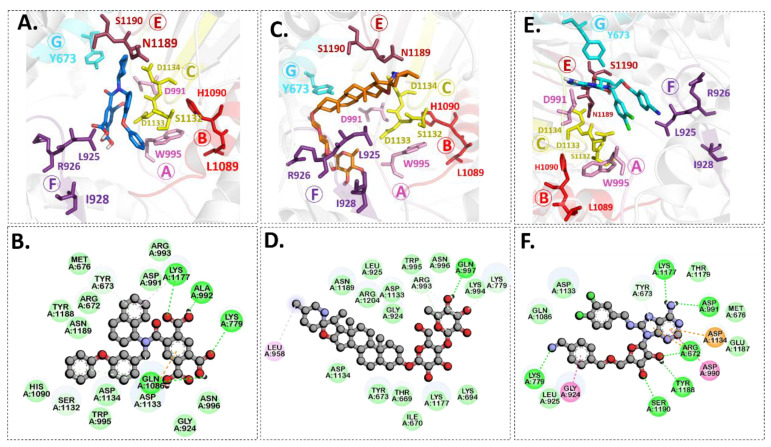
**Binding modes and interaction mechanisms of novel L protein inhibitors.** (**A**) Close-up view into binding mode of (**A**) A-317491, (**C**) Khasianine, and (**E**) VER155008. The main residues involved in the formation of the active site within the structurally conserved RdRp motifs shown in stickes. 2D interaction analysis of (**B**) A-317491, (**D**) Khasianine, and (**F**) VER155008.

Figure 4 shows the binding modes and 2D interaction mechanisms of top docked compounds. The 2D plot shows that A-317491 is involved in hydrogen bonding with several residues, such as His1090 and Gln1086 within motif E, Asp991, Ala992, Trp995, and Asn996 within motif A, and Lys 1177 within motif D, with a binding energy score of −8.7 kcal/mol (Figure 4B). Khasianine formed hydrogen bonding with a Gln997 side chain with a binding score of −7.1 kcal/mol (Figure 4C), while VER155008 was involved in making hydrogen bonds with residues Arg672, Lys779, Asp991, Lys1177, Tyr1188, and Ser1190 with a binding score of −7.8 kcal/mol (Figure 4D).

**Figure 5:**
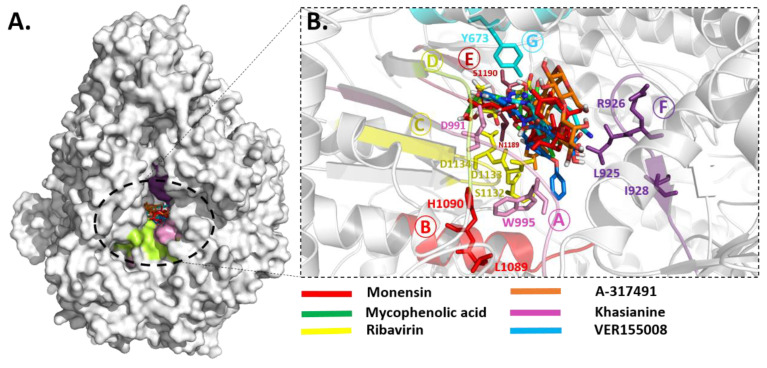
**Binding modes of compounds**. (**A**) Molecular surface representation for the inhibitory binding pattern of all ligands. (**B**) Close-up view into the binding mode of all compounds within the active site of RdRp core. The main residues involved in the formation of the active site within the structurally conserved RdRp motifs that are shown in stickes.

Notably, both known inhibitors and the identified compounds occupied the binding site pockets within the RdRp core with a similar interaction pattern (Figure 5). Both control drugs and selected compounds formed hydrogen bonds with functionally conserved aspartates of motif C, namely, Asp 1133 and the conserved divalent cation binding residue, Asp991 [24]. Our screened nucleoside compounds displayed higher binding energies and interacting profiles than Monensin, mycophenolic acid, and Ribavirin. Most ligands tended to bind the residues belonging to the PA-C-Like domain and the RdRp core domain. Since both these domains are actively involved in forming active site chamber and vRNA binding. Previously published data have proposed that RdRp and PA-C-Like domains facilitate the template-directed RNA synthesis in SFTSV-L protein by providing the NTP entry into the catalytic chamber [62]. Overall, both these domains and endo N domain are critical for product synthesis and the safest release in the cell [53]. These findings suggested that the compounds identified in our study could be more useful candidates for RVFV drug therapy. Previously, a number of studies on RdRp have identified several leads using various computational and experimental techniques. The compounds revealed to interact with RdRp binding pocket residues reported herein [86,87]. However, RVFV RdRp has not been explored much, and the current study is a pioneer work in this regard. 

### 2.4. ADME and Toxicity Prediction Analysis

Predicting ADME profiles using an in silico approach has long been proven to be a reliable method of determining a compound’s pharmacokinetic properties. Evaluating lead compounds’ ADME properties is a major challenge in the drug development [88]. Because of poor toxicity and pharmacokinetic properties, most drugs fail to pass the drug development process. The development of high-throughput and fast ADMET profiling assays has aided the detection of active lead compounds during early drug discovery [89]. Swiss ADMET and ADMETlab 2.0 were used to predict the absorption, distribution, metabolism, excretion, and toxicity (ADME) studies of the top three compounds, and their results are presented in Table 2. Gastro-intestinal absorption (GI) and blood-brain barrier (BBB) permeation indicate drug absorption and distribution of drug molecules [71]. One of the primary factors optimising drug discovery is information about drug distribution via BBB [90]. According to Table 2 results, all compounds show low gastro-intestinal absorption and no BBB permeation. The compounds cannot cross the blood-brain barrier (blood-brain barrier negative), so their consumption is not linked to the onset of neurological disorders. The absorption of the compounds was further revealed by caco-2 permeability values ranging from –6.019 to –5.727 log unit. A permeability of >−5.15 log unit in the ADMETlab 2.0 server indicates optimal caco-2 absorption. Oral bioavailability is frequently viewed as crucial in determining the drug-likeness of active compounds as therapeutic agents [91]. Furthermore, a variety of cytochromes (CYPs) regulate drug metabolism, with CYP2C19, CYP1A2, CYP2C9, CYP3A4, and CYP2D6 being critical for the biotransformation of drug molecules. The ability of a drug to inhibit or act as a substrate of the cytochrome P450 (CYP450) subfamily determines its therapeutic action [92]. A-317491 is an inhibitor of CYP2C9 while being a non-inhibitor and a non-substrate of other isoforms, while Khasianine is a non-inhibitor and a non-substrate of all isoforms, and VER155008 is an inhibitor of CYP3A4 and a non-inhibitor and non-substrates of other isoforms. Besides, p-glycoprotein inhibitors reduce the bioavailability of drugs that are known to be transported by it [93]. Except for Khasianine, all of the compounds in our analysis are inhibitors and negative substrates of p-glycoprotein, which explains the good absorption profile of the compounds. All of the compounds studied were nontoxic in terms of AMES toxicity. Following that, the safety profile of the three compounds was assessed by conducting toxicity prediction studies with an online tool: ProTox-II. This server classified substances into six toxicity classes (1–6), with class one being the most lethal and toxic, with an estimated lethal dose (LD50) of ≤5, and class six designating non-toxicity of the compound with an LD50 > 5000. A-317491 falls in class five with an LD50 value of 2500 mg/kg, while Khasianine falls in class four with an LD50 value of 500 mg/kg, and VER155008 falls in class six with an LD50 value of 7000 mg/kg. The findings strongly support the ability of the compounds studied to act as drugs against RVFV. 

### 2.5. MD Simulation

To understand the structural–functionality relationship of the target protein, MD simulations are essential in computer-aided drug design (CADD) [21]. MD simulations provide detailed biomolecule dynamical structural information and surface wealth of protein-ligand interactions and energetic data. MD simulations have been very successful in recent years for optimizing the docked hits [24,25,45,94,95] and related studies [23,52,95,96,97]. This data set can guide novel drug design, making MD simulation a valuable tool in modern drug discovery.

#### 2.5.1. Root Mean Square Deviation (RMSD)

**Figure 6:**
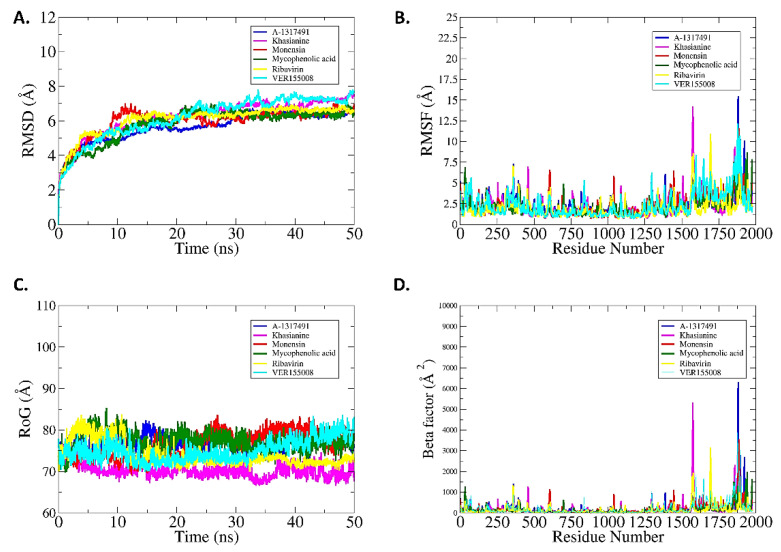
**MD-Simulation studies of ligand bounded complexes.** (**A**) Root mean square deviation. (**B**) Root mean square fluctuations (endoN domain 25–205 aa; PA-C-like domain 296–762 aa; RdRp core 763–1345 aa; PB2-N-like domain 1346–1571 aa; arm domain two spans 1615–1696 and 1811 aa; CBD domain 1697–1810 aa; and C-terminal domain 1933–2049 aa). (**C**) Radius of Gyrations. (**D**) Beta-factor analysis, each ligand is represented with different color such as A-1317491 (blue), Khasianane (pink), Monensin (Red), Mycophenolic acid (dark green), Ribavirin (Yellow), and Ver155008 (Cyan).

MD simulations of 50 ns were performed for the top three complexes and controls to elucidate compound binding stability and extract receptors/compound structural information that is important in the binding, and that may be altered to improve binding conformation and, ultimately, a compound affinity for the target biomolecule [95]. The RMSD is a frequently applied analysis to measure the structural similarity between superimposed proteins; smaller RMSD corresponds to similar structures and vice versa. The RMSD of each complex was calculated as carbon alpha deviations by superimposing 50,000 snapshots over the initial reference structure versus time. The RMSDs of the top three compounds were: A-317491-L (maximum, 6.76 Å; mean, 5.64 Å), VER155008-L (maximum, 7.82 Å; mean, 6.20 Å), and Khasianine-L (maximum, 7.67 Å; mean, 6.20 Å), while the RMSDs of the control drugs were: Monensin-L (maximum, 7.02 Å; mean, 5.95 Å), Mycophenolic acid-L (maximum, 7.24 Å; mean, 5.80 Å), and Ribavirin-L (maximum, 6.92 Å; mean, 6.0 Å) (Figure 6A). All the systems depicted uniform RMSD patterns, and no prominent peak was observed throughout the length of simulation time. The RMSD from the initial simulation time can be seen continuously for all complexes and converged at 10–15 ns. This is rightly possible because of the sudden exposure of the systems to a dynamic environment. This forces the receptor enzyme loops to acquire a more stable conformation. After 10 ns the systems can be witnessed in a more stable behavior until the end of the simulation time. The RdRp has been explored in SARS-CoV-2 for dynamics in the presence of a variety of drug molecules. The studies have found several leads that experience stable conformational dynamics and showed significant intermolecular stability with enzyme’s active pockets [98,99,100]. However, RdRp from RVFV is not investigated so far, making the findings of the current study interesting for experimentalists to test them in in vivo and in vitro validation. 

#### 2.5.2. Root Mean Square Fluctuation (RMSF) Analysis

The residual flexibility and stability of complexes were further computed. The mean RMSF for A-317491-L is 2.38 Å, VER155008-L is 2.37 Å, Khasianine-L is 2.23 Å, Monensin-L is 2.31 Å, mycophenolic acid-L is 2.16 Å, and Ribavirin-L is 1.98 Å. These values indicate a high level of agreement on intermolecular stability. Overall, a high rate of fluctuations was observed starting from residue 1250 to onward, and Khasianine-L and A-317491-L were among the ligands showing a high tendency to fluctuate (Figure 6B). It was observed that part of the RdRp core, PB2-N-like domain, CBD domain, and the C-terminal domain comprise a large percentage of flexible loops, forcing these segments to behave more dynamically. This may be a natural mechanism of the protein to confer some flexibility for mediating the proper accommodation of the substrate/ligand molecule inside the pocket and accomplish the catalytic mechanism. Moreover, the predicted binding site residues and their corresponding structural motifs were stable within a ~2 Å fluctuation in the presence of ligands. The less flexibility in the RdRp core region indicated that the ligands adopted a more stable conformation and established consistent interactions with the important surrounding residues. 

#### 2.5.3. Radius of Gyration (Rg)

Furthermore, Rg analysis was used to assess structural equilibrium and protein compactness over the simulation time. Rg is employed to investigate whether the binding of the compounds affects the overall structural stability of the receptor enzyme. Lower Rg indicates the tight packing of the enzyme atoms and less effect of the compounds on the enzyme structure upon binding, thus presenting the stable nature of the subject complex. The Rg of the complexes follows; A-317491-L (maximum, 82.41 Å; mean, 76.76 Å), Khasianine-L (maximum, 76.76 Å; mean,70.16 Å), VER155008-L (maximum, 83.67 Å; mean, 75.05 Å), Monensin-L (maximum, 83.64 Å; mean, 76.43 Å), Mycophenolic acid-L (maximum, 85.40 Å; mean, 77.29 Å), and Ribavirin-L (maximum, 83.76 Å; mean, 74.09 Å) (Figure 6C). All six complexes are quite stable and remain compact. These Rg results complement the RMSD result in interpreting the docked complex stability.

#### 2.5.4. B-Factor Analysis

B-factors were also derived from simulation trajectories to probe highly mobile regions of the complexes. The B-factor monitors the thermal motion of protein atoms, side chains, and whole regions. The B-factor is commonly used to identify internal protein motions, probing rigid and flexible regions important in proteins/enzyme functionality. The average values of the B-factor for the top three complexes A-317491-L, VER155008-L Khasianine-L, and control drugs Monensin-L, Mycophenolic acid-L, and Ribavirin-L were: 201.68 Å^2^, 210.62 Å^2^,187.20 Å^2^, 183.67 Å^2^, 155.86 Å^2^, and 139.35 Å^2^, respectively (Figure 6D). It demonstrates that these complexes have good stability throughout the 50 ns simulation period. From the simulation results, it can be concluded that all the studied systems are structurally stable, and the intermolecular interactions remained strong throughout the simulation time. 

### 2.6. MMGBA/PBSA Analysis

Binding free energies were estimated using MMPBSA and MMGBSA techniques to understand better the compounds’ binding potential with the L protein. Table 3 shows the detailed binding energies of the complexes [101,102]. As all binding interactions are energetically favorable, stable complexes are formed. Gas-phase energy dominates the system energy in all complexes, with van der Waals playing a significant role, while electrostatic energy plays a minor role. This reflects that both van der Waals and electrostatic interactions are key in intermolecular stability. The polar solvation energy is unfavorable in binding, whereas the nonpolar energy appears to be favourable in complex equilibration. The complexes’ MMGBSA net binding-energy rankings were as follows: A-317491-L > VER155008-L > Khasianine-L > Mycophenolic acid-L > Monensin-L > Ribavirin-L. The complexes’ MMPBSA net binding-energy rankings were as follows: A-317491-L > Khasianine-L > VER155008-L > Monensin-L > Mycophenolic acid-L > Ribavirin-L. These results indicate that our compounds have high binding free energies than control drugs.

## 3. Materials and Methods

### 3.1. Homology Modeling 

Drug discovery relies heavily on homology modeling, with current efforts resulting in unprecedented accuracy models, even with low sequence identity [32,103,104,105,106,107]. Because L protein lacked a crystal structure, homology modeling was required to determine target structure elucidation. The L protein sequence was obtained from the UniProt database (P27316 (L_RVFVZ)) to perform homology modeling [108]. Sequence to Structure alignment was performed using PSI-BLAST against Protein Data Bank (PDB) to find suitable templates for the three-dimensional (3D) structure prediction [109,110]. The initial alignment between the target and the template was generated using the ALIGN2D module. The final model was built using a restrained-based approach in Modeller.v9.11 with the most-fitted template and secondary structural information obtained by manual curation after superimposition between all generated models and the template [111]. Modeller’s secondary structure module was used to model the final structure using the extracted spatial secondary structure restraints. 

### 3.2. Structure Validation

Accurate evaluation of the 3D model is considered one of the core elements of computational structure prediction. The emergence of rapidly endorsed and highly efficient approaches for structure evaluation has paved new ways to assess the quality of newly designed models [112,113]. Ramachandran plots are a quick and easy way to evaluate the quality of a 3D structure. The Ramachandran plot was used to determine the energetically permissible and prohibited phi (ϕ) and psi (ψ) dihedral angles of amino acid residues [114]. The 3D structure was further analyzed by PDBsum [56]. PDBsum is a database that summarises the contents of 3D macromolecular structures. In addition, the predicted 3D structure was refined and validated by using 50 ns MD simulations.

### 3.3. Target Protein Preparation

3D structure of L protein predicted from Modeller and validated with 50 ns MD simulations were prepared using the AMBER 18 program [115]. The ff14SB force field [116] was utilized to parameterize amino acids. AMBER18’s tleap module was used to add complementary hydrogen atoms missed by crystallography. Energy minimization of the target protein was performed first for 1000 steepest descent steps and then for 500 conjugate gradient steps, allowing the step size to be 0.02 Å. After MD clustering, the most stable conformation was selected from the cluster (with RMSD < 1 Å) for further processing.

### 3.4. Compound Preparation

The Selleckchem Nucleoside Analogue Library (https://www.selleckchem.com/screening/Nucleoside-Analogue-Library.html, accessed on 15 February 2021) was used to identify molecules with the highest binding affinity to the L-protein. The library composed of 156 natural compounds was downloaded in 3D SDF format. A recent study reported Monensin, Mycophenolic acid, and Ribavirin as potent inhibitors of RVFV-L protein [79]. To get an insight into the mechanism of the binding of known inhibitors, these three potent inhibitors were docked as controls in the present study. These drugs were retrieved from the PubChem database (https://pubchem.ncbi.nlm.nih.gov, accessed on 15 February 2021) in 3D SDF format [117]. 

### 3.5. Structure-Based Virtual Screening

The AutoDock Vina implicated in the PyRx (version 0.8) was used to perform the virtual screening of the compounds against the target protein [118,119]. The control drugs and chemical library of 156 compounds in SDF format were imported in the Open-babel program of PyRx for further energy minimization, followed by conversion of all the ligands into AutoDock PDBQT format. Further, the top three ligands were subjected to docking against the L protein of RVFV using AutoDock Vina. The grid box was set to cover the entire binding site within the core chamber of RdRp with box_size of (x = 20, y = 20, z = 20 Å) and dimensions of (x = 122.005, y = 105.326, and z = 137.824). Dockings were run in triplicates to ensure absolute consistency of the results. The docked solutions were clustered using an RMSD of 1 Å. The 3D structural alignment, visualization and analysis, and docking figures production were carried out using Discovery Studio 2019 software [120], PyMOL [121], and UCSF Chimera 1.14 [122].

### 3.6. ADME and Toxicity Prediction Analysis

The analysis of the pharmacological activity of drug candidates is a critical step in drug discovery [93]. Therefore, in silico prediction of pharmacophore properties of active compounds is critical for accelerating the drug development process. Absorption, distribution, metabolism, excretion, and toxicity (ADMET) are the most plausible drug-like properties to evaluate virtual hits. ADME analysis was carried out by submitting the compounds’ canonical simplified molecular-input line-entry system (SMILES) to online servers; ADMETlab 2.0 [123] and Swiss ADME [124]. Acute oral toxicity was predicted by the Protox II web server [125].

### 3.7. MD Simulations

AMBER18 was used to perform MD simulations of the docked solutions [115]. The same MD simulation protocol was adopted in the current study as described in previous studies [22,94,126,127,128]. The Antechamber package of AmberTools was employed to generate the general AMBER force field (GAFF) parameters for the studied ligands using AM1–BCC charge definitions. Each top complex was explicitly solvated with water molecules, and then counter ions were added to create a neutral system. A water box with a thickness of 12 Å was created using the TIP3P solvent model to encircle the complex [129,130]. The complex was simulated using periodic boundary conditions, with electrostatic interactions modeled using the particle mesh Ewald procedure [131]. For nonbounded interactions, a threshold value of 8 Å was defined during the procedure. Water molecules were minimized for 500 cycles, and then the entire system was minimized for 1000 rounds. The temperature of each system was then steadily increased to 300 K. Solutes in the first phase were restrained for 50 ps during equilibration of counterions and water molecules, while protein side chains were relaxed afterwards. At 300 K and 1 atm, a 50 ns MD simulation was performed, and coordinate trajectories were collected every 2 ps during the simulation under the NPT ensemble. While the SHAKE algorithm [132] constrained covalent and hydrogen bonds, Langevin dynamics [133] was used to control the system temperature. The original structure was used as a reference, and AMBER’s CPPTRAJ [134,135] was used to generate an RMSD (root mean square deviation) plot to assess the system’s MD simulation convergence [136]. The structural flexibility of ligands was calculated using ligand RMSD. The radius of gyration (RoG) was analyzed to determine the complex’s compactness and three-dimensional packing. RMSF reflects the root mean square averaged distance between an atom and its average geometric position [137]. The Term β--factor, which is closely related to the RMSF, measures the spatial displacement of atoms around their mean positions, as well as a thermal and local vibrational movement [138].

### 3.8. MMGBA/PBSA Analysis

AMBER18 MM/PBSA and MM/PBSA methods were used to estimate the binding free energy (G binding) of the complexes [101,139]. The method has been well-documented in our previous studies, implemented in AMBER 18. The free energy difference was calculated using 100 snapshots from simulated trajectories at regular intervals. The total binding free energy is calculated as a sum of the molecular mechanics binding energy (ΔE_MM_) and solvation free energy (ΔG_sol_) as follows:

ΔE_MM_ = ΔE_int_ + ΔE_ele_ + ΔE_vdw_

ΔG_sol_ = ΔG_p_ + ΔG_np_

ΔG_total_ = ΔE_MM_ + ΔG_sol_

ΔG_bind_ = ΔE_MM_ + ΔG_sol_ − TΔS

In these equations, ΔE_MM_ is further divided into internal energy (ΔE_int_), electrostatic energy (ΔE_ele_), and van der Waals energy (ΔE_vdw_), and the total solvation free energy (ΔG_sol_) is contributed by the sum of polar (ΔG_p_) and non-polar (ΔG_np_) components. ΔG_bind_ is the free energy of binding of the ligand evaluated after entropic calculations (TΔS). We excluded entropic term, TΔS, from the calculation due to its intensive computational cost [101,102,140,141]. These methods have been well documented [101,128,140] and considered reliable end-point binding free energy estimations [102,141]. 

## 4. Conclusions

A combination of virtual screening and molecular docking analysis was used to identify inhibitors of RVFV L protein in the present study. The 3D structure of the RVFV-L enzyme was predicted, and the findings could aid researchers working on RVFV drug development. The Selleckchem Nucleoside Analogue Library (156 compounds) was screened, and the top three compounds, A-317491, VER155008, and Khasianine exhibit a high binding affinity compared to the control drugs chosen that may inhibit RVFV-L enzyme activity and hence virus replication. Compounds also show good pharmacodynamic and pharmacokinetic profiles based on toxicity and ADME studies. Overall, the results reveal that all of the compounds fit into the same binding pocket of the protein, suggesting that they could be good therapeutic candidates for RVFV. However, further in-vivo and in-vitro experiments are required to convert these potential inhibitors into clinical drugs. We anticipate that the findings of this study will be useful in the future for developing and exploring novel natural anti-RVFV therapeutic agents.

## Figures and Tables

**Table 1:** Binding energy and binding residues of docked complexes.

Docked Complex	Chemical Structure	Binding Energy kcal/mol	Binding Residues (Amino Acid ID)	
			Van der Waals	Hydrogen Bonds
**A-317491**	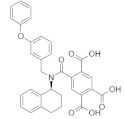	−8.7	673, 1132, 1133	1090, 995, 1134, 925, 1086, 924, 996, 779, 992, 1177, 993, 991, 676, 672, 1188, 1189
**Monensin**	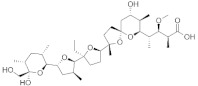	−8.5	676, 1187, 989, 1179, 1135, 991, 673, 995, 694, 1134, 1189	672, 990, 1133, 992
**VER155008**	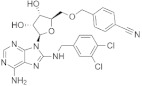	−7.8	925, 1189, 1187, 676, 989, 1179, 1178, 673, 1133, 1086	779, 1190, 1188, 672, 991, 1177
**Khasianine**	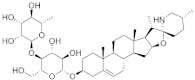	−7.1	1189, 1204, 924, 1133, 925, 993, 995, 996, 994, 779, 694, 1177, 670, 669, 673, 1134	997
**Mycophenolic acid**	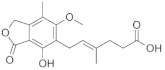	−7.0	1181, 1180, 1127, 1126, 1183, 751, 667, 985, 1171, 1128, 668, 1170	1197, 984
**Ribavirin**	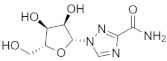	−6.7	1135, 1188, 990, 989, 1178, 1177, 676, 1189	1134, 757, 1190, 672, 991, 1133

**Table 2:** Predicted ADME and Toxicity properties of identified nucleoside analogs. The probability of each parameter is depicted. **BBB**: blood–brain barrier; **CYP450**: cytochrome P450.

Parameters	Compounds		
	A-317491	Khasianine	VER155008
**Absorption**			
BBB	No	No	No
GI absorption	Low	Low	Low
Caco-2 permeability	−6.019	−5.356	−5.727
Human oral bioavailability	0.56	0.17	0.17
Log P	5.296	2.723	2.136
TPSA (Å^2^)	141.44	179.56	166.21
**Metabolism**			
P-glycoprotein substrate	No	Yes	No
P-glycoprotein inhibitor	No	No	No
CYP450 2C9 substrate	No	No	No
CYP450 2D6 substrate	No	No	No
CYP450 3A4 substrate	No	No	No
CYP450 1A2 inhibitor	No	No	No
CYP450 2C9 inhibitor	Yes	No	No
CYP450 2D6 inhibitor	No	No	No
CYP450 2C19 inhibitor	No	No	No
CYP450 3A4 inhibitor	No	No	Yes
**Toxicity**			
AMES Toxicity	Non-toxic	Non-toxic	Non-toxic
Carcinogens	Non-carcinogenic	Non-carcinogenic	Non-carcinogenic
Acute oral toxicity	2500 mg/kg	500 mg/kg	7000 mg/kg

**Table 3:** Binding free energy of docked complexes. The energy values are given in kcal/mol units.

Energy Component	Average	Standard Error of Mean	Average	Standard Error of Mean	Average	Standard Error of Mean	Average	Standard Error of Mean	Average	Standard Error of Mean	Average	Standard Error of Mean
	A-1317491		Khasianine		Monensin		Mycophenolic Acid		Ribavirin		VER155008	
**MM-GBSA**												
** ΔE_vdw_**	−65.37	2.72	−54.45	4.89	−53.83	3.23	−48.81	5.00	−26.20	3.57	−51.64	3.87
** ΔE_ele_**	−56.86	6.92	−257.10	17.11	−80.72	5.55	−30.21	4.83	−155.72	7.14	−87.45	11.03
** ΔG_p_**	74.46	6.80	275.40	16.39	100.29	4.76	42.30	4.30	158.69	5.83	98.08	8.68
** ΔG_np_**	−7.10	0.19	−6.51	0.31	−6.92	0.39	−5.04	0.18	−3.75	0.26	−5.72	0.29
** ΔE_MM_**	−122.23	7.68	−311.55	18.54	−134.56	6.12	−79.03	8.37	−181.93	7.33	−139.09	9.68
** ΔG_sol_**	67.36	6.68	268.88	16.23	93.36	4.68	37.26	4.21	154.93	5.86	92.35	8.74
** ΔG_total_**	−54.87	2.75	−42.66	4.16	−41.19	2.89	−41.76	4.96	−26.99	4.216	−46.73	3.41
**MM-PBSA**												
** ΔE_vdw_**	−65.37	2.72	−54.45	4.89	−53.83	3.23	−48.81	5.00	−26.20	3.57	−51.64	3.87
** ΔE_ele_**	−56.86	6.92	−257.10	17.1	−80.72	5.55	−30.21	4.83	−155.72	7.14	−87.45	11.03
** ΔG_p_**	95.85	7.89	288.12	16.80	114.98	8.24	57.57	5.53	166.11	6.22	118.11	7.49
** ΔG_np_**	−4.73	0.10	−4.99	0.13	−5.52	0.16	−3.18	0.08	−2.34	0.07	−4.44	0.12
** ΔE_MM_**	−122.2	7.68	−311.55	18.54	−134.56	6.12	−79.03	8.37	−181.93	7.33	−139.09	9.68
** ΔG_sol_**	91.12	7.84	283.12	16.76	109.45	8.19	54.39	5.53	163.77	6.20	113.66	7.49
** ΔG_total_**	−31.11	4.89	−28.42	4.30	−25.10	5.39	−24.64	6.16	−18.16	5.23	−25.42	5.87

## Data Availability

The data presented in this study are available within the article and supplementary material.

## Supplementary Materials

The following supporting information can be downloaded at: https://www.mdpi.com/article/10.3390/ph15060659/s1, Figure S1. Sequence alignment of RVFV L protein with the templates (Structure of the alpha-Synuclein (PDB ID: 6I42 and severe fever with thrombocytopenia syndrome virus L protein (PDB ID: 6Y6K). Figure S2. Structural superposition of the RVFV L protein model with the templates. The RMSD values between the model and 6I42 and 6Y6K are 0.179 and 1.165 Å, respectively. Table S1. Reverse template comparison of RVFV L protein model with the representative structures in PDB using ProFunc reverse template search program. Table S2. Virtual Screening result of Selleckchem Nucleoside Analogue Library (compounds with binding affinity > −1.5 kcal/mol) against model of RVFV-L RdRp core.
