# Supplementary material for: Structural Elucidation of Rift Valley Fever Virus L Protein towards the Discovery of Its Potential Inhibitors
Source: Pharmaceuticals (Basel). 2022 May 25;15(6):659. doi: 10.3390/ph15060659 (PMC9228520; doi:10.3390/ph15060659)
Supplement: Supplementary file 1 [file pharmaceuticals-15-00659-s001.zip › pharmaceuticals-1717850-supplementary.pdf]

Supporting Information

Structural elucidation of Rift Valley Fever Virus L protein towards the discovery of its potential inhibitors

Mubarak A. Alamri[1,†], Muhammad Usman Mirza[2,†], Muhammad Muzammal Adeel[3,†], Usman Ali Ashfaq[4], Muhammad Tahir ul Qamar[*,4], Farah Shahid[4], Sajjad Ahmad[5], Eid A. Alatawi[6], Ghadah M. Albalawi[7,8], Khaled S Allemailem[7,*] and Ahmad Almatroudi[7]

1 Department of Pharmaceutical Chemistry, College of Pharmacy, Prince Sattam Bin Abdulaziz University, Al-Kharj, Saudi Arabia; m.alamri@psau.edu.sa
2 Department of Chemistry and Biochemistry, University of Windsor, Windsor, Canada; mumirza@uwindsor.ca
3 3D Genomics Research Center, College of Informatics, Huazhong Agricultural University, Wuhan, China; m.muzammal.adeel@outlook.com
4 Department of Bioinformatics and Biotechnology, Government College University Faisalabad, Faisalabad 38000, Pakistan; usmancemb@gmail.com (U.A.A.); tahirulqamar@gcuf.edu.pk (M.T.Q.); farahshahid24@gcuf.edu.pk (F.S.)
5 Department of Health and Biological Sciences, Abasyn University, Peshawar, Pakistan; sajjad.ahmad@abasyn.edu.pk
6 Department of Medical Laboratory Technology, Faculty of Applied Medical Sciences, University of Tabuk, Tabuk 71491, Saudi Arabia; eid.alatawi@ut.edu.sa
7 Department of Medical Laboratories, College of Applied Medical Sciences, Qassim University, Buraydah 51452, Saudi Arabia; galbalawi080@gmail.com (G.M.A.); k.allemailem@qu.edu.sa (K.S.A.); aamtrody@qu.edu.sa (A.A.)
8 Department of Laboratory and Blood Bank, King Fahd Specialist Hospital, Tabuk 47717, Saudi Arabia.

† These authors contributed equally to this study.
* Correspondence: tahirulqamar@gcuf.edu.pk (M.T.Q.); k.allemailem@qu.edu.sa (K.S.A.)

```
RVFV_L         sequencequery----MDSILSK--QLVDKTGFVRVPIKHFDCTMLTLALPTFDVSKMV  54
6L42_0_0       --structureL-FIRSTX-------------------------------GLPDLDVTVDA  27
6Y6K_1_0       --structureYKFIRSTX-------------------------------GLPDLDVTVDA  28
                 . . *   *             **                        .** :**:  .

RVFV_L         DRITIDFNLDDIQGASEIGSTLLPSM-SIDVEDMANFVHDFTFGHLADKTDRLLMREFPM  113
6L42_0_0       TGVTVDIGAVP-DSAS---------------SEAYKINHDFTFSGLSKTTDRRLSEVFPI  71
6Y6K_1_0       TGVTVDIGAVP-DSASQLGSSINAGLITIQLSEAYKINHDFTFSGLSKTTDRRLSEVFPI  87
                 :*:*:.    :.**               .:  :: *****. *:..*** * . **:

RVFV_L         MNDGFDHLSPDMIIKTTSGVYNIVEFTNFRGDERGAFQAAM-IKLAKYEVPCENRS---Q  169
6L42_0_0       THDGSDGMTPDVIHTRLDGTIVVVEFTTTRSHNIGGLEAAYRTKIEKYRDPISRRVDIME  131
6Y6K_1_0       THDGSDGMTPDVIHTRLDGTIVVVEFSTTRSHNIGGLEAAYRTKIEKYRDPISRRVDIME  147
                :** * ::**:* .  .*.  :***:. *..: *.::**   *: **. * ..*    :

RVFV_L         GRTVVLYVVSAYRAWCMVYLELERTLKQREMVYRYRLALSVMDELRTLFP--ELSSTDEE  227
6L42_0_0       NPRVFFGVIVVSSGGVLSNMPLTQD-EAEELMYRFCIANEIYTKARSMDADIELQKSEEE  190
6Y6K_1_0       NPRVFFGVIVVSSGGVLSNMPLTQD-EAEELMYRFCIANEIYTKARSMDADIELQKSEEE  206
               .  *.: *: .  .  :  : * :  : .*::**: :* .:  : *::    **..::**

RVFV_L         LGKTERELPAMVSSIQINWSVTESVFPPFSREMFDRFRSSPPDSEYITRIVSRCLINSQE  287
6L42_0_0       LEAISRAL-SFFSLFEPNIERVEGTFPNSEIEMLEQFLSTPADVDFITKTLKAKEVEAYA  249
6Y6K_1_0       LEAISRAL-SFFSLFEPNIERVEGTFPNSEIKMLEQFLSTPADVDFITKTLKAKEVEAYA  265
               *   .* * ::.* :: * . .*..**  . :*:::* *:* * ::**: :.   :::

RVFV_L         KLINSSFFAEGNDKALRFSKNAEECSLAVERALNQYRAEDNLRDLNDHKSTIQLPPWLSY  347
6L42_0_0       DLCDSHYLKPEKTIQERLEINRCEAIDKTQDLLAGLHARSN-KQTSLNRGTVKLPPWLPK  308
6Y6K_1_0       DLCDSHYLKPEKTIQERLEINRCEAIDKTQDLLAGLHARSN-KQTSLNRGTVKLPPWLPK  324
               .* :* ::   :    *:. *  *.   .:  *    :*..* :: . ::.*::*****

RVFV_L         HDVDGKDLCPLQGLDVRGDH-PMCNLWREVVTSANLEEIERMHDDAAAELEFAFGS---K  403
6L42_0_0       PSSESIDIKTDSGFGSLMDHGAYGELWAKCLLDVSLGNVEGVVSDP--ELDIA-------  359
6Y6K_1_0       PSS-----------------GAYGELWAKCLL----------------------------  339
                .                     :** : :                          ***

RVFV_L         GQARE-RNRYHRVHLNMGSDVLVYIAALGVNGKKHK---ADTLVQQMRDRSKQPFSP---  456
6L42_0_0       DTPKEAKITYRRFK---PALSSSARQEFSLQG----------------------LSPFLD  394
6Y6K_1_0       ----------------------------------------------SHETLSP---FLD  349
                                                    ***

RVFV_L         DHDVITYLNF-S-LHALVTCGQQMRTCT--ALSLVIRDSVGSPEDSSAILVRKGFHEIIT  512
6L42_0_0       VEDIGDFLTF-NNLLADSRYGDE-SVQR--AVSILL-------EKASAMQDTELTHALND  443
6Y6K_1_0       VEDIGDFLT-FNNLLTDSRYGDESIQ---RAVSILL-------EKASAMQDTELTHALND  398
                .*:  :*.  . * :    *::     * *:*:::       *.:**:   :  * :

RVFV_L         EHYKFMGSRIGHGCQMVSLIGAELSASVKQHVKPNYFVIKRLLGSGIFLLIKPTSSKSHI  572
6L42_0_0       SFKRNLSSNVVQWSLWVSCLAQELASALKQHCRAGEFIIKKLKFWPIYVIIKPTKSSSHI  503
6Y6K_1_0       SFKRNLSSNVVQWSLWVSCLAQELASALKQHCRAGEFIIKKLKFWPIYVIIKPTKSSSHI  458
               .. : :.*.: : .  ** :. **::::*** : . *:**:*    *:::****.*.***

RVFV_L         FVSFALSALAGPLISPLPG-FSSPTKMLGILLVTDFVSYKLSKLTNLCKCVSLMESSFSF  631
6L42_0_0       F--YSLGIRKADVTRRLTGRVFSETIDAGEWELTEFKSLKTCKLTNLVNLPCTMLNSIAF  561
6Y6K_1_0       F--YSLGIRKADVTRRLTGRVFSDTIDAGEWELTEFKSLKTCKLTNLVNLPCTMLNSIAF  516
               *  ::*.   . :   * * . * *   *   :*:* * * .***** :  . * .*::*

RVFV_L         WAEAFGIQAGTLVGDFVPRSSDSAAMDASYMGKLSLLTLLEDKAATEELQTIARYIIMEG  691
6L42_0_0       WREKLGV---------APWLVRKPCSELREQVGLTFLISLEDKSKTEEIITLTRYTQMEG  612
6Y6K_1_0       WREKLGV---------APWLVRKPCSELREQVGLTFLISLEDKSKTEEIITLTRYTQMEG  567
               * * :*:         .*    . . :      *::*  ****: ***: *::**  ***

RVFV_L         FVSPPEIPKPHKMTSKFPKVLRSELQVYLLNCLCRTIQRIAGEPFILKKKDGSISWGGMF  751
6L42_0_0       FVSPPMLPKPQKMLGKLDGPLRTKLQVYLLRKHLDCMVRIASQPFSLIPREGRVEWGGTF  672
6Y6K_1_0       FVSPPMLPKPQKMLGKLDGPLRTKLQVYLLRKHLDCMVRIASQPFSLIPREGRVEWGGTF  627
               ***** :***:** .*:   **::******.     : ***.:** *  ::* :.*** *

RVFV_L         NPFSGRPLLDMQPLISCCYNGYFKNKEEETEPSSLSGMYKKIIELEHLRPQSDAFLGYKD  811
6L42_0_0       HAISGRST-NLENMVNSWYIGYYKNKEESTELNALGEMYKKIVEMEEDKPSSPEFLGWGD  731
6Y6K_1_0       HAISGRST-NLENMVNSWYIGYYKNKEESTELNALGEMYKKIVEMEEDKPSSPEFLGWGD  686
               : :***   ::: ::.. * **:*****.** .:*. *****:*:*. :*.*  ***: *

RVFV_L         PELPRMHEFSVSYLKEACNHAKLVLRSLYGQNFMEQIDNQIIRELSGLTLERLATLKATS  871
```

```
6L42_0_0      TDSPKKHEFSRSFLRAACSSLEREIAQRHGRQWKQNLEERVLREIGTKNILDLASMKATS   791
6Y6K_1_0      TDSPKKHEFSRSFLRAACSSLEREIAQRHGRQWKQNLEERVLREIGTKNILDLASMKATS   746
               : *: **** *:*: **.  :  : . :*::: ::::::::**:.  .:  **::****

RVFV_L        NFNENWYVYKDVADKNYTRDKLLVKMSKYASEGKSLAIQKFEDCMRQIESQGCMHICLFK   931
6L42_0_0      NFSKDWELYSEVQTKEYHRSKLLEKMATLIEKGVMWYIDAVGQAWKAVLDDGCMRICLFK   851
6Y6K_1_0      NFSKDWELYSEVQTKEYHRSKLLEKMATLIEKGVMWYIDAVGQAWKAVLDDGCMRICLFK   806
              **.::* :*.:*  *:* *.*** **:.  .:*    *: . :. : : .:***:*****

RVFV_L        KQQHGGLREIYVMGAEERIVQSVVETIARSIGKFFASDTLCNPPNKVKIPETHGIRARKQ   991
6L42_0_0      KNQHGGLREIYVMDANARLVQFGVETMARCVCELSPHETVANPRLKNSIIENHGLKSARS   911
6Y6K_1_0      KNQHGGLREIYVMDANARLVQFGVETMARCVCELSPHETVANPRLKNSIIENHGLKSARS   866
              *:***********.*: *:**  ***:**.: ::   :*:.**  * .* *.**::: :.

RVFV_L        CKGPVWTCATSDDARKWNQGHFVTKFALMLCEFTSPKWWPLIIRGCSMFTKKRMMMNLNY   1051
6L42_0_0      LGPGSININSSNDAKKWNQGHYTTKLALVLCWFMPAKFHRFIWAAISMFRRKKMMVDLRF   971
6Y6K_1_0      LGPGSININSSNDAKKWNQGHYTTKLALVLCWFMPAKFHRFIWAAISMFRRKKMMVDLRF   926
                    .  :*:**:******:.**:**:** *   *:  :*  . *** :*:**::*.:

RVFV_L        LKILDGHRELDIRDDFVMDLFKAYHGEAEVPWAFKGKTYLETTTGMMQGILHYTSSLLHT   1111
6L42_0_0      LAHLS--------DPFREAMTDAFHGNREVSWMDKGRTYIKTETGMMQGILHFTSSLLHS   1023
6Y6K_1_0      LAHLSS-------DPFREAMTDAFHGNRDVSWMDKGRTYIKTETGMMQGILHFTSSLLHS   979
              *  *.        * *   : .*:**: :* *  **:**::* *********:******:

RVFV_L        IHQEYIRSLSFKIFNLKVAPEMSKSLVCDMMQGSDDSSMLISFPADDEKVLTRCKVAAAI   1171
6L42_0_0      CVQSFYKSYFVSKLKEGYMGE-SISGVVDVIEGSDDSAIMISIRPKSDMDEVRSRFFVAN   1082
6Y6K_1_0      CVQSFYKSYFVSKLKEGYMGE-SISGVVDVIEGSDDSAIMISIRPKSDMDEVRSRFFVAN   1038
                *.: :*  .. ::     * * * * *:::*****:::**:   ..:   .*.:. .*

RVFV_L        CFRMKKELGVYLAIYPSEKSTANTDFVMEYNSEFYFHTQHVRPTIRWIAACCSLPEVETL   1231
6L42_0_0      LLHSVKFLNPLFGIYSSEKSTVNTVYCVEYNSEFHFHRHLVRPTLRWIAASHQISETEAL   1142
6Y6K_1_0      LLHSVKFLNPLFGIYSSEKSTVNTVYCVEYNSEFHFHRHLVRPTLRWIAASHQISETEAL   1098
               ::  * *.  :.** *****.** : :******:** : ****:*****. .: *.*:*

RVFV_L        VARQEEASNLMTSVTEGGGSFSLAAIIQQAQCTLHYMLMGMGVSELFLEYKKAVLKWNDP   1291
6L42_0_0      ASRQEDYSNLLTQCLEGGASFSLTYLIQCAQLLHHYMLLGLCLHPLFGTFMGMLISDPDP   1202
6Y6K_1_0      ASRQEDYSNLLTQCLEGGASFSLTYLIQCAQLLHHYMLLGLCLHPLFGTFMGMLISDPDP   1158
              .:***: ***:*.  ***.****: :** **   ****:*: :  **  :   ::.  **

RVFV_L        GLGFFLLDNPYACGLGGFRFNLFKAITRTDLQKLYAFFMKKVKGSAARDWADEDVTIPET   1351
6L42_0_0      ALGFFLMDNPAFAGGAGFRFNLWRACKTTDLGRKYAYYFNEI------------YRALDA   1250
6Y6K_1_0      ALGFFLMDNPAFAGGAGFRFNLWRACKTTDLGRKYAY-----------------------   1195
              .*****:***  .* .******::* . *** : **:

RVFV_L        CSVSPGGALILSSSLKWGSRKKFQKLRDRLNIPENWIELINENPEVLYRAPRTGPEILLR   1411
6L42_0_0      TS---GGTLSHSVMVYWGDRKKYQALLNRMGLPEDWVEQIDENPGVLYRRAANKKELLLK   1307
6Y6K_1_0      ------------------------------------------------------------   1195

RVFV_L        IAEKVHSPGVVSSLSSGNAVCKVMASAVYFLSATIFEDT----GRPEFNFLEDSKYSLLQ   1467
6L42_0_0      LAEKVHSPGVTSSLSKGHVVPRVVAAGVYLLSRHCF-FSSSIH--G-----STQKASLIK   1359
6Y6K_1_0      -----------------HVVPRVVAAGVYLL-----------------------------   1209
                               :.* :*:*:.**:*

RVFV_L        --KMAAYSGFHGFNDMEPEDILFLFPNIEELESLDSIVYNKGEIDIIPRVNIRDATQTRV   1525
6L42_0_0      LLMMSSISAMKHGGSLNPNQERMLFPQAQEYDRVCTLLEEVEH------VRERNIVRSRI   1413
6Y6K_1_0      ------------GGSLNPNQERMLFPQAQEYDRVCTLLEEVEHLTGKFVVRERNIVRSRI   1257
                           ..::*::  :***: :* : : ::: :  .      *. *: .::*:

RVFV_L        TIFNEQKNLRTSPEKLVSDKWFGTQKSRIGKTTFLAEWEKLKKIVKWLEDAPEATLAHTP   1585
6L42_0_0      DLFQEPVDLRCKAEDLVSEVWFGLKRTKLGPRLLKEEWDKLRASFAWLSTDPSETLRDGP   1473
6Y6K_1_0      DL----------------------------------------------------------   1259
               :

RVFV_L        LNNHIQVRNFFARMESKP-RTVRITGAPVKKRSGVSKIAMVIRDHFSRMGHLRGVEDLAG   1644
6L42_0_0      FLSHVQFRNFI-------------------------------------------SLDN   1488
6Y6K_1_0      ------------------RSVRLL------------------------------GAP---   1268
                                 *

RVFV_L        FTRSVSAEILKHFLFCILQGPYSESYKLQLIYRVLSSVSNVEIKESDGK-----------   1693
6L42_0_0      QERLESISILKHVLFMVLNGPYTEEYKLEMIIEAFSTL----------------------   1526
```

```
6Y6K_1_0      -----TIS---QVVRM----NFFPGFSLE-------------------------------  1285
                   : .   :.:       :   :.*:                     ***********

RVFV_L        ------------------TKTNLIGILQRFLD--GDHVVPIIEEMGAGTVGGFIKRQQSK  1733
6L42_0_0      -------------------RTMTLCLLSN-------SILDQIERAQSGTLGGFSKPQ-KT  1559
6Y6K_1_0      ------------------------------------------------------------  1285
              ******************              **

RVFV_L        V--VQNKVVYYGVGIWRGFMDGY-QVHLEIENDIGQPPRLRNVTTNCQSSPWDLSIPIRQ  1790
6L42_0_0      FIRPGGGVGYKGKGVWTGVME-DTHVQILIDGD-GTSNWLEEIRLSSDARLYDVIESIRR  1617
6Y6K_1_0      ------------------------------------------------------------  1285


RVFV_L        WAEDMGVTNNQDYSSKSSRGARYWMHSFRMQG-PSKPFGCPVYIIKGDMSDVIRLRK---  1846
6L42_0_0      LCDDLGINNRVASAYRGHCMVR--LSGFKIKPASRTD-GCPVRI----MERGFRIRELQN  1670
6Y6K_1_0      ------------------------------------------------------------  1285


RVFV_L        -EEVEMKVRGSTLNLYTKHHSHQDLHILSYTASDNDLSPGIFKSISDEGVAQALQLFERE  1905
6L42_0_0      PDEVKMRVRGDILNLSVTIQEGRVMNILSYRPRDTDISESAAAYLWSN--RDLFSFGKKE  1728
6Y6K_1_0      ------------------------------------------------------------  1285


RVFV_L        PSNCWVRCESVAPKFISAILEICEGKRQIKGINRTRLSEIVRICSESSLRSKVGSMFSFV  1965
6L42_0_0      PSCSWICLKT--------ASVLLANDRKTQGIDNRAMGNIFRDCLEGSLR-KQGLMRSKL  1779
6Y6K_1_0      ------------------------------------------------------------  1285


RVFV_L        ANVEEAHDVDYDA--LMDLMIEDAKNNAFSHVVDC-IELDVSGPYEMES-FHGRSTLTCT  2021
6L42_0_0      TEMVEKNVVPLTTQELVDILEEDID---FSDVI-AV-ELS-EGSLDIESIFDGAPIL---  1830
6Y6K_1_0      ------------------------------------------------------------  1285


RVFV_L        PSTILIRTYTFYLTLHQTMISVQAFQVILDEGVLLIALVNNYLRGSKANCWVRCESVAPK  2081
6L42_0_0      ------------------------------------------------------------  1830
6Y6K_1_0      ------------------------------------------------------------  1285


RVFV_L        FISAILEICEGK-RQIK-GINRT-RLSEI-VEFVLNLPKIKSRIYVLICRQCHGANFPPI  2137
6L42_0_0      ------------------------------------------------------------  1830
6Y6K_1_0      ------------------------------------------------------------  1285
                          *    *     *     *

RVFV_L        SVRRLMLEDIASVARRLIIVASFGS*  2162
6L42_0_0      ------------------------*-  1830
6Y6K_1_0      ------------------------*-  1285
```

Figure S1. Sequence alignment of RVFV L protein with the templates (Structure of the alpha-Synuclein (PDB ID: 6I42 and severe fever with thrombocytopenia syndrome virus L protein (PDB ID: 6Y6K)**.**

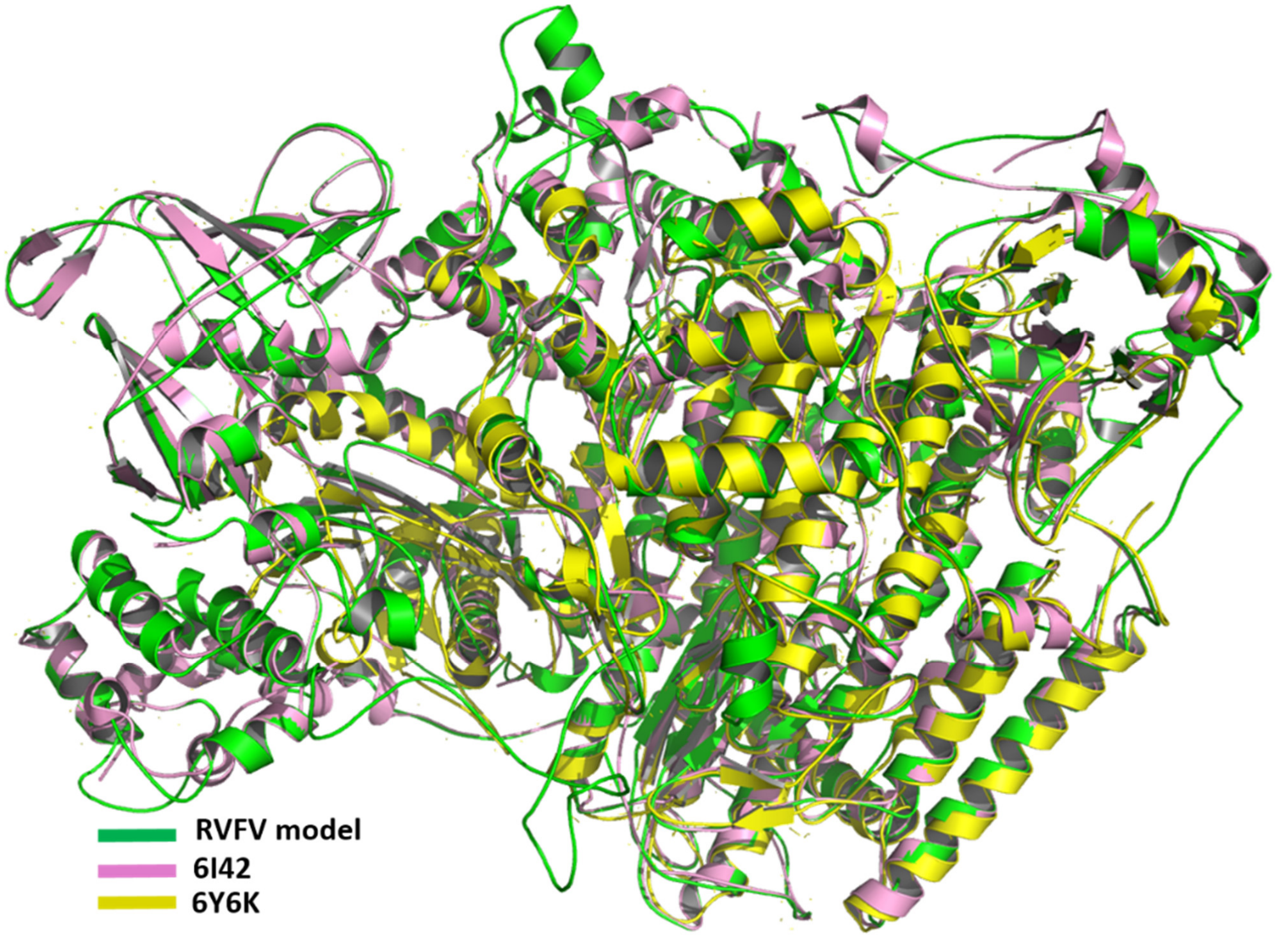

Figure S2. Structural superposition of the RVFV L protein model with the templates. The RMSD values between the model and 6I42 and 6Y6K are 0.179 and 1.165 Å, respectively.

Table S1. Reverse template comparison of RVFV L protein model with the representative structures in PDB using ProFunc reverse template search program.

Hit no.[a]	Matched PDB entry	E-value	RMSD (Å)[b]	Similarity score	Seq id (%)	Structural attributes	
						Longest fitted segment[c]	Structural similarity (%)
1	6l42	0	0.26	702.3	32.78%	1756/1756	99.9

Note: [a] Hit number corresponds to the best ranked hit from PDB (against a representative subset of structures in the PDB).
[b] Root-mean-square-deviation between Cα between templates (in PDB) and RVFV L protein model.
[c] Represents structural significant superimposition between query and template structures, which starts from a sliding window of seven residues lengthwise is slide along the alignment and the C-alpha atoms of the equivalenced residues in both structures through superimposition. The fit gives an rmsd below the cut-off of 3.0Å along the length of the window size, which extends until that cut-off.

Table S2. Virtual Screening result of Selleckchem Nucleoside Analogue Library (compounds with binding affinity > -1.5 kcal/mol) against model of RVFV-L RdRp core.

No.	Ligand	Binding Energy (kcal/mol)
1	VER155008	-9.6
2	S9012_Khasianine	-9.1
3	A-317491	-8.7
4	S9430_Tomatidine	-8.7
5	LLY-283	-8.5
6	Cilostazol	-8.4
7	S5461_Cimicifugoside	-8.4
8	etravirine	-8.4
9	Guanosine.	-8.3
10	NADIDE	-8.3
11	S5402_Dasabuvir(ABT-333)	-8.3
12	Fludarabine	-8.1
13	S4994_Methylcobalamin	-8.1
14	SGC_0946	-8.1
15	S5109_Ademetionine	-8
16	S5246_Entecavir.cdx_uff_E=616.44	-8
17	CGS-21680_hydrochloride	-7.9
18	Dapivirine	-7.9
19	S5358_Regadenoson	-7.9
20	S9007_2-Deoxyguanosine_monohydrate	-7.9
21	6688	-7.8
22	Dibutyryl-cAMP	-7.8
23	S3641_Osalmid	-7.8
24	S3757_2'-Deoxyinosine	-7.8
25	S5215_Abacavir	-7.8
26	8-Bromo-cAMP	-7.7
27	Clevudine	-7.7
28	Capecitabine_(Xeloda)	-7.6

**29**	N6-methyladenosine	-7.6
**30**	Blasticidin_S_hydrochloride	-7.5
**31**	Puromycin_dihydrochloride	-7.5
**32**	Sofosbuvir(PSI-7977)	-7.5
**33**	CELLCEPT_(mycophenolate_mofetil)	-7.4
**34**	EPZ004777	-7.4
**35**	Fludara_(Fludarabine_Phosphate)	-7.4
**36**	Ribothymidine _Thymine_riboside	-7.4
**37**	Rilpivirine	-7.4
**38**	S3610_Cordycepin	-7.4
**39**	S9366_5-Adenylic_acid.	-7.4
**40**	DIDANOSINE_(didanosine)	-7.3
**41**	Doxifluridine.cdx_uff_E=510.10	-7.3
**42**	S5259Nicotinamide_Mononucleotide	-7.3
**43**	S5374_5'-Cytidylic_acid	-7.3
**44**	S9368_ADP	-7.3
**45**	Spongouridine_1-beta-D-Arabinofuranosyluracil	-7.3
**46**	TRIFLURIDINE_(trifluridine)	-7.3
**47**	VIDAZA_(azacitidine)	-7.3
**48**	valganciclovir_hydrochloride	-7.3
**49**	6-Thio-dG	-7.2
**50**	Arranon_(Nelarabine)	-7.2
**51**	DEPOCYT_(cytarabine)	-7.2
**52**	Inosine	-7.2
**53**	S4984_2-Cyclouridine	-7.2
**54**	S5009_Brivudine	-7.2
**55**	S5582_Cytarabine_hydrochloride	-7.2
**56**	S5678_trans-Zeatin-riboside	-7.2
**57**	S9369_Xanthosine_Dihydrate	-7.2
**58**	untitled	-7.2
**59**	Floxuridine	-7.1
**60**	Gemcitabine_hydrochloride	-7.1
**61**	Ribavirin	-7.1
**62**	S3737_Cangrelor_Tetrasodium	-7.1
**63**	TYZEKA_(telbivudine)	-7.1
**64**	Clofarabine	-7
**65**	Cyclocytidine_hydrochloride	-7
**66**	Penciclovir	-7
**67**	S9275_Isoguanosine	-7
**68**	Stavudine	-7
**69**	VIRA-A_(vidarabine)	-7
**70**	S5505_2-deoxyuridine	-6.9
**71**	Uridylic_acid	-6.9
**72**	aicar	-6.9
**73**	decitabine_(2-deoxy-5-azacytidine)	-6.9
**74**	Acyclovir	-6.8
**75**	Adenosine	-6.8

**76**	Cladribine	-6.8
**77**	5-Iodotubercidin	-6.7
**78**	S4685_Efavirenz	-6.7
**79**	TAS-102	-6.7
**80**	Tubercidin	-6.7
**81**	Ganciclovir	-6.6
**82**	HIVID_(zalcitabine)	-6.6
**83**	NEVIRAPINE_(nevirapine)	-6.6
**84**	NSC23766	-6.6
**85**	6853	-6.5
**86**	Azaguanine-8	-6.5
**87**	Emtricitabine	-6.5
**88**	Ftorafur	-6.5
**89**	GEMCITABINE_(gemcitabine)	-6.5
**90**	5-Methyldeoxycytidine	-6.4
**91**	5110	-6.4
**92**	Mizoribine	-6.4
**93**	S5362_Citicholine	-6.4
**94**	Zidovudine	-6.4
**95**	Bromodeoxyuridine	-6.3
**96**	S4803_Thymidine	-6.3
**97**	Uridine	-6.3
**98**	Cytidine	-6.2
**99**	EPIVIR_(lamivudine)	-6.2
**100**	Idoxuridine	-6.2
**101**	S5307_2-deoxy-2-fluoro-2-C-methyluridine	-6.2
**102**	SQ22536	-6.2
**103**	Zebularine	-6.1
**104**	vistonuridine	-6.1
**105**	LM-3264_Triapine	-6
**106**	5143_uff_E=60.88	-5.6
**107**	Adrucil_(Fluorouracil)	-5.5
**108**	Hypoxanthine	-5.5
**109**	S4785_Nicotinamide_N-oxide	-5.3
**110**	S3651_4-Amino-5-imidazolecarboxamide	-5.1
**111**	Hydroxyurea	-4.1
**112**	S5016_Isoprinosine	-3.8
**113**	s4515_Ademetionine_Ademetionine_disulfate_tosylate	-3.7
**114**	2416	-3.6
**115**	5383	-3.6
**116**	Entecavir_hydrate	-1.8
**117**	S9351_2-Deoxyadenosine_monohydrate	-1.8
**118**	S5284_Adenosine_5-monophosphate_monohydrate	-1.7
**119**	S5297_Vidarabine_monohydrate	-1.7
